# Diagnosing gastrointestinal infections based on cycle threshold cut-offs of PCR

**DOI:** 10.1128/spectrum.01234-24

**Published:** 2024-12-19

**Authors:** Rania Karam, Peter Kechker, Shifra Ken-Dror, Avi Peretz, Maya Azrad

**Affiliations:** 1 W. Hirsch Regional Microbiology Laboratory, Clalit Health Services, Haifa, Israel; 2 Azrieli Faculty of Medicine, Bar Ilan University, Safed, Israel; 3 Clinical Microbiology Laboratory, Tzafon Medical Center (affiliated with Azrieli Faculty of Medicine, Bar Ilan University, Safed, Israel), Poriya, Israel; London Health Sciences Centre, London, Ontario, Canada

**Keywords:** gastrointestinal infections, culture, molecular methods, cycle threshold, *Shigella*, *Salmonella*

## Abstract

This study compared the performance of molecular vs stool culture assays for gastrointestinal infection (GII) detection, with focus on defining cycle threshold (Ct) cut-off values for positive culture results. A total of 6,000 records of patients with suspected GII between October 2022 and February 2023 and registered at Clalit HealthCare Services in Haifa, Israel, were reviewed. Stool samples were collected from all patients with suspected GII. PCR was performed with the Seegene Allplex GI-Bacteria (I) assay kit. PCR-positive samples were cultured on bacteria-specific agar media. Out of 356 PCR-positive samples, 196 (55.1%) were culture-positive. Significant differences were noted between the mean Ct of culture-positive vs culture-negative samples for *Shigella* spp. (*P* < 0.0001), *E. coli* O157 (*P* = 0.0001), and *Campylobacter* spp. (*P* = 0.004). *Shigella* had the lowest Ct cutoff (27.14). Negative culture results for PCR-positive samples may result from low bacterial load. At the same time, false-positive PCR results may exist. Thus, PCR result should be considered along with clinical presentation and with Ct value consideration.

**IMPORTANCE:**

GII diagnostic procedures have shifted from traditional- to molecular-based assays, which may increase missdiagnosis due to the high PCR sensitivity and false positives. This study suggests to consider a Ct threshold for each pathogen in order to reduce inaccurate diagnosis. Alternatively, culture should be performed for PCR-positive samples.

## INTRODUCTION

Gastrointestinal infections (GII) are one of the most prevalent infections worldwide and constitute a major health concern. In most cases, the infection self-resolves with supportive care, including fluid and electrolyte supplementation (1). However, antibiotic treatment may be required, especially in cases of severe infections or in high-risk patients, such as the elderly or immunocompromised individuals (2).

Accurate diagnosis of GII is important for informing medical decisions concerning the administration of antibiotics. Additionally, rapid identification is essential in cases of foodborne and waterborne outbreaks (1). Diagnosis of GII typically involves a combination of clinical evaluation and laboratory tests. Stool culture, the gold standard laboratory method for diagnosing bacterial GII, is a valuable tool due to its non-invasive nature, high specificity, and cost-effectiveness as compared to other procedures, such as endoscopy and colonoscopy. Yet, the method suffers from low sensitivity and range of detection and long turnaround time and requires skilled personnel for interpretation (3). In recent years, new molecular-based multiplex assays enabling simultaneous detection of multiple pathogens have been introduced (4–6). These assays offer several advantages over traditional methods, including rapidity, which may improve treatment outcomes, and increased sensitivity and specificity (5). They are expected to accurately detect low burdens of organisms without making the result a false positive. These assays also enable the detection of pathogens whose cultivation from stool samples is challenging or requires special culture conditions, such as in the case of *Clostridioides difficile* (5). Their drawbacks include high cost and the need for specific instruments (6). Additionally, the detection of bacterial DNA does not necessarily indicate a current infection, as high positivity rates were found among asymptomatic individuals (7). Finally, the main disadvantage of molecular assays is their inability to assess antibiotic susceptibility, as they do not involve bacterial isolation. In an era with increased antibiotic resistance, particularly among GII pathogens, knowledge regarding antibiotic resistance is critical for optimizing treatments (8).

The current study aimed to compare the performance of molecular assay vs stool culture GII diagnosis, among 6,000 patients, registered at Clalit Health Care Services in Haifa, Israel. It also aimed to define a cycle threshold (Ct) cut-off value for the polymerase chain reaction (PCR) testing that could differentiate culture-positive and culture-negative samples. Furthermore, it was conducted to determine the prevalence of bacterial pathogens causing GII in north Israel.

## MATERIALS AND METHODS

### Study population

This study analyzed records of patients (age 0–100 years) registered in the Clalit Health Care Services (CHCS) in Haifa, Israel, with reported suspicion of GII between October 2022 and February 2023. CHCS is the largest state-mandated health medical organization in Israel, servicing 5.4 million members across the country, including 1.2 million in north Israel. Stool samples were collected as an integral part of routine medical care and transferred to the clinical laboratory within 24 h. Samples were collected in a sterile cup.

### Molecular identification of intestinal bacteria

Genomic DNA was extracted from stool samples using the STARMag Universal Cartridge kit (Seegene, Duesseldorf, Germany) on the Hamilton Microlab Starlet platform (Hamilton Company, Reno, Nevada, USA). The DNA was then analyzed by multiplex PCR with the Seegene Allplex GI-Bacteria (I) assay kit (Seegene, Seoul, South Korea), which identifies *Escherichia coli* (*E.coli*) O157, *Salmonella* spp., *Shigella* spp., and *Campylobacter* spp. PCR was conducted on a CFX96 instrument (Bio-Rad, Marnes-la-Coquette, France), under the following conditions: 50°C for 20 min, 95°C for 15 s, followed by 45 cycles of 10 s each, at 95°C, 1 min at 60°C, and 30 s at 72°C (9). Results were interpreted with the Seegene Viewer software.

### Stool cultures

PCR-positive samples were cultured on the medium suitable for the pathogen detected by PCR: HY-*Campylobacter* medium (Hy Laboratories, Rehovot, Israel), Sorbitol MacConkey agar (Hy Laboratories), *Salmonella*/*Shigella* Agar (Novamed, Jerusalem, Israel), and CHROMAGAR *Salmonella* Plus (Hy Laboratories). Agar plates were incubated at 37°C for 24 h, with the exception of the plate for *Campylobacter*, which was incubated at 42°C for 24 h, under microaerophilic conditions. Bacteria were initially identified based on the morphological characteristics of the colonies (color and shape) and then using the Vitek-MS MALDI-TOF technology system (BioMerieux, Marcy l'Etoile, France). The *Escherichia coli* O157 Latex Test kit (ThermoFisher Scientific, Oslo, Norway) was used for precise identification of *E. coli* O157 strain. Agglutination assays for *Shigella* spp. and *Salmonella enterica* spp. serotyping were performed with commercially available antisera from MAST ASSURE (Mast Group Ltd., Liverpool, UK).

### Statistical analysis

The Chi-squared test was used to compare the number positive results obtained with PCR vs stool culture. The optimal Ct cut-off (between negative- and positive-culture samples) was determined for each bacterial species to maximize sensitivity and specificity. A *t*-test was performed to compare the mean Ct of positive-culture samples with negative-culture samples. Analysis of variances (ANOVA) was used to compare variances across the mean Ct between different bacterial species. Statistical analysis was performed with SAS version 9.3 (SAS Institute, Cary, North Carolina). Statistical significance was defined as *P* < 0.05.

## RESULTS

A total of 6,000 patient records were analyzed. In 196 PCR-positive cases, cultures were positive, with 70 cases of *Campylobacter*, 8 cases of *E. coli* O157, 80 cases of *Shigella,* and 38 cases of *Salmonella*. The PCR analysis yielded 356 positive samples, with 113 cases of *Campylobacter*, 45 cases of *E. coli* O157, 151 cases of *Shigella,* and 47 cases of *Salmonella*. The Ct ranges were 20.12–38.92 for *Campylobacter*, 23.99–43.58 for *E. coli* O157, 15.01–42.57 for *Shigella,* and 19.96–39.78 for *Salmonella*. The maximum Ct values for positive cultures were 37.87 for *Campylobacter*, 31.96 for *E. coli* O157, 34.4 for *Shigella,* and 39.2 for *Salmonella*.

**Fig 1:**
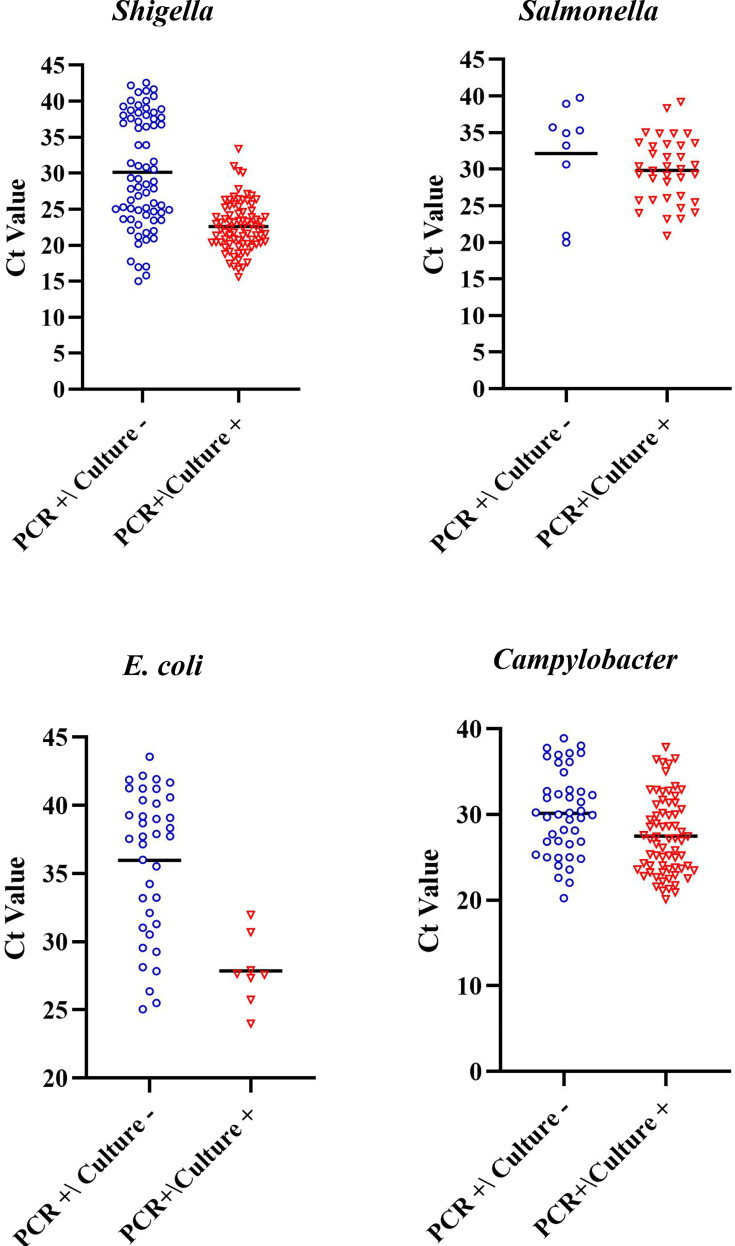
Ct values for samples from patients with a gastrointestinal infection with a positive vs negative culture sample. PCR-positive samples were cultivated on several agar media. Ct values of culture-positive samples were compared to the Ct values of culture-negative samples, for *Shigella*, *Salmonella*, *E. coli,* and *Campylobacter*.

Figure 1 presents a comparison between the Ct values of culture-positive vs culture-negative samples, for each pathogen. The mean Ct value (Ct = 30.13) of *Shigella* spp.-positive PCR samples with negative cultures was significantly higher than the mean Ct value (Ct = 22.59) of *Shigella* spp.-positive PCR samples with positive cultures (*P* < 0.0001) (Fig. 1; Table 1). Similarly, mean Ct values of 27.85 and 35.96 were recorded for *E. coli O157*-positive PCR samples with a positive vs negative culture, respectively (*P* = 0.0001). An identical trend was noted for C*ampylobacter* spp.-positive culture samples (mean Ct: 27.48) as compared to negative-culture samples (meant Ct: 30.16) (*P* = 0.004). In contrast, no statistically significant difference was observed between the Ct values of *Salmonella*-positive vs *Salmonella*-negative culture samples (Table 1). The optimal Ct cutoff was lowest (27.14) for *Shigella* and highest for *Salmonella* (34.91) (Table 2).

**TABLE 1:** Ct of culture-positive and culture-negative samples from patients with gastrointestinal infection

Bacteria	Mean Ct		*P*
	Positive culture	Negative culture	
*Campylobacter* spp.	27.48	30.16	0.004
*E. coli O157*	27.85	35.96	0.0001
*Salmonella*	29.84	32.16	0.224
*Shigella*	22.59	30.13	<0.0001

**TABLE 2:** Optimal Ct cutoff to distinguish between positive and negative culture samples

Bacteria	Optimal cutoff	Sensitivity	Specificity	*P*
*Campylobacter*	29.4	0.676	0.395	0.0036
*E. coli* O157	31.96	1	0.263	0.0001
*Salmonella*	34.91	0.917	0.444	0.0009
*Shigella*	27.14	0.938	0.423	<0.0001

Mean Ct values varied for the *Campylobacter* spp., but with no statistically significant difference (*Campylobacter coli*: 25.44 vs *Campylobacter jejuni*: 28.02; *P* = 0.061) (Table 3). In contrast, a significant difference in the mean Ct value was noted for samples carrying *Shigella flexneri* (28.17) vs *Shigella sonnei* (22.9) (*P* = 0.0006).

**TABLE 3:** Mean Ct values of positive samples according to species

Bacteria	Species	Mean Ct	*P*
*Campylobacter* spp.	*Campylobacter coli* *Campylobacter jejuni*	25.44 28.02	0.061
*Shigella* spp.	*Shigella flexneri* *Shigella sonnei*	28.17 22.9	0.0006

## DISCUSSION

The continuous pursuit to enhance the analytical sensitivity of clinical microbiology methods has brought many laboratories to replace some of their culture-based assays with molecular technologies (9). However, increased analytical sensitivity may be accompanied by lower “clinical” specificity and consequently, a reduced positive predictive value (10). This study compared the performance of culture-based vs molecular testing of clinical specimens from patients with suspected GII by culturing all PCR-positive samples. For all pathogens, PCR yielded higher detection rates as compared to culture. These findings corroborate previous studies that compared PCR and culture (8, 10) and were not surprising as PCR is known for its increased sensitivity (11). Cases of positive PCR, but negative culture can be explained by antibiotic or bismuth administration which may affect culture outcomes but not PCR (11). A previous study indicated that a considerable portion of *Campylobacter* infections may go undetected by the traditional culture method (12).

Many preanalytical and analytical factors can affect the viability of pathogens, which may lead to false negative culture (4). In the current study, the samples least missed by culture were *Salmonella* and *E. coli* 0157, which suggests that these pathogens are less sensitive to the preanalytical conditions. Specifically, the high cultivation rate of *Salmonella* may be associated with the enrichment step routinely used to specifically enhance *Salmonella* cultivation success (4).

Despite the improved sensitivity of PCR, clinical symptoms must also be considered, as a positive molecular result, as opposed to culture, does not guarantee pathogen viability; PCR may be positive in cases of prolonged shedding, asymptomatic carriage or in the late stages of infection. Therefore, clinical evaluation is mandatory in GII and samples should be collected from symptomatic patients only (1).

Significant differences were noted in the mean Ct of *Shigella*, *E. coli* O157-positive, and *Campylobacter* spp-positive samples as compared to their negative culture counterparts. Another study on *Campylobacter* also found a significantly lower mean Ct for PCR-positive-culture-positive samples compared to PCR-positive-culture-negative samples (*P* < 0.01) (13). In their analysis of stool samples of children aged 2–59 months in Zanzibar, Elfving et al. found the Ct of *Shigella* PCR-positive samples from symptomatic patients to be significantly lower than the Ct of *Shigella*-PCR-positive samples from asymptomatic controls (*P* < 0.0001) (14). In contrast, no significant differences were observed between the Ct values of symptomatic vs asymptomatic patients carrying *Campylobacter* or *Salmonella*. Similar to the present results, a study which analyzed 1,056 stool samples by both PCR and culture reported on significantly lower Ct in PCR- and culture-positive samples as compared to samples with positive PCR but negative culture (*P* < 0.001). These differences were observed for all tested pathogens, i.e., *Campylobacter* (*P* < 0.001), *Salmonella* (*P* = 0.02), and *Shigella* (*P* = 0.02) (4). Generally, lower Ct values reflect higher bacterial loads. The presented findings and those reported by others suggest that low bacterial load may result in cultivation failure. As mentioned above, such cases may represent asymptomatic carriage, prolonged shedding, or end of infection. Consequently, combination of PCR and clinical assessment provides for a more accurate diagnostic outcome.

The mean Ct of PCR-positive-culture-positive samples was highest for *Salmonella* and lowest for *Shigella*. A similar finding was reported by others (4). Variations in mean Ct values were also noted across *Campylobacter* and *Shigella* species and may be due to differences in bacterial loads. Alternatively, the sensitivity of molecular assays may differ for various pathogens. Further studies should be performed to investigate this point. Another important point is that not all commercial PCR kits provide a Ct value, and therefore, identifying positive samples by Ct cannot be performed in all laboratories.

The current study used two-step confirmation of GII, i.e., all PCR-positive samples were confirmed by culture. Growing bacteria from PCR-positive samples can provide valuable information relating to antibiotic susceptibility, strain typing, and epidemiology, which can guide clinical management and public health interventions. Additionally, confirming PCR-positive results with culture can help validate the accuracy of molecular diagnostic assays and identify potential false-positive results. However, these benefits must be weighed against the additional time, labor, and resources associated with specimen culturing, especially in settings where rapid diagnosis and treatment are paramount. Furthermore, the interpretation of culture results from PCR-positive samples should be cautiously approached due to potential contamination or overgrowth of commensal flora. Ultimately, the decision to pursue bacterial culture should be guided by clinical judgment, laboratory capacity, and the specific objectives of the diagnostic investigation. Future research could explore the cost-effectiveness and clinical impact of incorporating culture confirmation into routine molecular diagnostic workflows, aiming to optimize diagnostic strategies for GII while balancing resource constraints and patient care priorities.

## Data Availability

The data sets generated during and/or analyzed during the current study are available from the corresponding author on reasonable request. The data used and/or analyzed during the current study are available from the corresponding author on reasonable request.
